# Associations of Longitudinal Fetal Growth Patterns With Cardiometabolic Factors at Birth

**DOI:** 10.3389/fendo.2021.771193

**Published:** 2021-12-09

**Authors:** Jia-Shuan Huang, Qiao-Zhu Chen, Si-Yu Zheng, Rema Ramakrishnan, Ji-Yuan Zeng, Can-Peng Zhuo, Yu-Mian Lai, Ya-Shu Kuang, Jin-Hua Lu, Jian-Rong He, Xiu Qiu

**Affiliations:** ^1^ Division of Birth Cohort Study, Guangzhou Women and Children’s Medical Center, Guangzhou Medical University, Guangzhou, China; ^2^ Paediatrics School, Guangzhou Medical University, Guangzhou, China; ^3^ Department of Obstetrics and Gynecology, Guangzhou Women and Children’s Medical Center, Guangzhou Medical University, Guangzhou, China; ^4^ National Perinatal Epidemiology Unit, Nuffield Department of Population Health, University of Oxford, Oxford, United Kingdom; ^5^ Department of Women and Child Health Care and Provincial Key Clinical Specialty of Woman and Child Health, Guangzhou, China; ^6^ Nuffield Department of Women's and Reproductive Health, University of Oxford, Oxford, United Kingdom

**Keywords:** fetal growth, cord blood, birth cohort, insulin, glucose, lipid

## Abstract

**Background:**

Birth weight is associated with cardiometabolic factors at birth. However, it is unclear when these associations occur in fetal life. We aimed to investigate the associations between fetal growth in different gestational periods and cord blood cardiometabolic factors.

**Methods:**

We included 1,458 newborns from the Born in Guangzhou Cohort Study, China. *Z*-scores of fetal size parameters [weight, abdominal circumference (AC), and femur length (FL)] at 22 weeks and growth at 22–27, 28–36, and ≥37 weeks were calculated from multilevel linear spline models. Multiple linear regression was used to examine the associations between fetal growth variables and *z*-scores of cord blood cardiometabolic factors.

**Results:**

Fetal weight at each period was positively associated with insulin levels, with stronger association at 28–36 weeks (*β*, 0.31; 95% CI, 0.23 to 0.39) and ≥37 weeks (*β*, 0.15; 95% CI, 0.10 to 0.20) compared with earlier gestational periods. Fetal weight at 28–36 (*β*, −0.32; 95% CI, −0.39 to −0.24) and ≥37 weeks (*β*, −0.26; 95% CI, −0.31 to −0.21) was negatively associated with triglyceride levels, whereas weight at 28–36 weeks was positively associated with HDL levels (*β*, 0.12; 95% CI, 0.04 to 0.20). Similar results were observed for AC. Fetal FL at 22 and 22–27 weeks was associated with increased levels of insulin, glucose, and HDL.

**Conclusions:**

Fetal growth at different gestational periods was associated with cardiometabolic factors at birth, suggesting that an interplay between fetal growth and cardiometabolic factors might exist early in pregnancy.

## Introduction

Fetal life is the period that an individual is most susceptible to environmental influences due to high plasticity of cells and organs (1, 2). Disturbed intrauterine development can have long-term health implications. According to the developmental origins of health and disease hypothesis, both fetal undernutrition and overnutrition may cause permanent changes to glucose–insulin and lipid metabolism and lead to cardiometabolic diseases in childhood and adulthood such as obesity, cardiovascular diseases, hyperlipidemia, and type 2 diabetes mellitus (1, 2).

Cardiometabolic markers in cord blood reflect the intrauterine metabolic environment and conditions of the fetus and may be an indicator of long-term changes in metabolic functions after birth. Previous studies (3–5), including ours (6), have shown that birth weight is associated with cardiometabolic markers in cord blood, such as insulin, glucose, and lipids. For example, birth weight *z*-score was positively related to cord blood insulin levels (6). In addition, compared with appropriate-for-gestational-age (AGA) infants, small-for-gestational-age (SGA) infants tend to have higher cord blood triglycerides (TG) and lower insulin, high-density lipoprotein cholesterol (HDL), total cholesterol, and low-density lipoprotein cholesterol (LDL) levels (7, 8). In contrast, large-for-gestational-age (LGA) infants have higher cord blood insulin level (9). All these studies have used birth weight as a proxy of fetal growth. However, birth weight is a summary indicator and does not reflect the longitudinal pattern of fetal growth. It is unclear when the association between fetal growth and cardiometabolic markers appears during prenatal period. Also, different fetal biometric parameters represent different dimensions of fetal growth. For instance, abdominal circumference (AC) is considered an indicator of body fat storage and is most predictive of birth weight, whereas femur length (FL) reflects fetal skeletal growth (10). These measures may have different implications for metabolic changes in the fetus. However, no study has examined the associations of different fetal biometric parameters and cardiometabolic markers in cord blood.

In the present study, we investigated the relationship between fetal growth measures (fetal weight, AC, and FL) at different gestational periods and cord blood cardiometabolic factors. The findings of our study may provide insights into the critical period for fetal programming of cardiometabolic health in childhood and adulthood.

## Methods

### Study Population

This study was part of the Born in Guangzhou Cohort Study—BIGCS, a prospective study led by Guangzhou Women and Children’s Medical Center (GWCMC) that aimed to investigate the influence of environmental, genetic, and social factors on the health of pregnant women and the next generation. The protocol and cohort profile of BIGCS have been previously published (11). Briefly, BIGCS pregnant women are invited to participate when they undergo the first routine prenatal check-ups at GWCMC if they are less than 20 weeks of gestation, planned to give birth at GWCMC, and intended to stay in Guangzhou for ≥3 years after delivery. These women and their children are followed up at multiple time points to collect epidemiological and clinical information and biospecimens. BIGCS has been approved by the Ethics Committee of GWCMC. Written consent has been obtained from all participants.

The eligibility criteria for the current analysis include 1) availability of cord blood samples, 2) availability of maternal fasting blood samples at 14–27 weeks of gestation and data on 2-h 75-g oral glucose tolerance test (OGTT) of the mother, 3) no severe disease before pregnancy (e.g., type 1 or type 2 diabetes, hypertension, kidney diseases), and (4) availability of data on maternal demographic characteristics. This analysis included 1,744 mother–child pairs who were randomly selected from those eligible. Mother–child pairs with no fetal ultrasound growth data (*n* = 212) or covariates (*n* = 74) were excluded. This study finally included 1,458 pairs of mothers and children.

### Fetal Growth Assessment

Data on fetal biometric measures, including biparietal diameter, head circumference, AC, and FL, were obtained through ultrasound scanning and extracted from the hospital electronic system. Fetal weight was estimated (EFW) using the INTERGROWTH-21st formula based on AC and FL (12). On average, each pregnant woman had three (range, 1–7) ultrasound examinations for fetal growth. An ultrasound examination is routinely performed in the first trimester to confirm gestational age based on crown–rump length. For pregnant women who do not attend antenatal care in the first trimester, gestational age is assessed in early second trimester based on fetal biometrics, including bilateral parietal diameter, head circumference, AC, FL, or cerebellar diameter (13, 14). Well-trained and licensed ultrasonographers in GWCMC conducted these ultrasound measurements. As our previous study shows (15), the mean error of EFW estimation ([EFW − birth weight]/birth weight × 100%) is low (about 4%), and the correlation between two consecutive measurements of fetal biometric parameters performed within a week was high (*r* > 0.95). These results indicate good quality of the ultrasound data.

Based on our previous study (15) and other studies (16–18), a multilevel linear spline model was used to evaluate the patterns of fetal growth in different gestational periods. Briefly, a fractional polynomial model was first used to determine the best-fitting curve for fetal size (including EFW and birth weight) by gestational age for the whole sample. Two knots (i.e., 28 and 37 weeks) were then selected to divide the entire gestation period into three intervals: “mid-pregnancy” (22–27 weeks), “early-third trimester” (28–36 weeks), and “late-third trimester” (≥37 weeks). We then fitted multilevel linear spline models to estimate fetal growth during each period. The resulting random effects from the models denote individual deviations from average size in early pregnancy (i.e., 22 weeks) and the average growth velocities in the three gestational intervals, which were converted into sex-specific *z*-scores before further analyses. More information on the modeling process can be found in our previous report (15). The same modeling approach was applied to generate AC and FL *z*-scores based on ultrasound measurements. Fetuses were also classified into “slow,” “normal,” and “fast” growth if they had a *z*-score ≤–1.28 (i.e., 10th percentile), –1.28 to 1.28, and >1.28 (i.e., 90th percentile), respectively. These cutoff values are equivalent to those for SGA, AGA, and LGA at birth which are commonly used in clinical practice.

### Measurement of Cardiometabolic Markers in Cord Blood

Trained midwives collected venous umbilical cord blood using tubes with EDTA anticoagulants. The blood was centrifuged within 24 h. Plasma samples were obtained and stored at −80°C until analysis. A third-party medical laboratory assessed the levels of cardiometabolic factors in cord blood, including insulin, glucose, total cholesterol, HDL, LDL, and TG. Specifically, insulin levels (μIU/ml) were measured using the Roche Immunology Analyzer (cobas 8000 e602, Roche, Basel, Switzerland), and glucose (mmol/L), total cholesterol (mmol/L), HDL (mmol/L), LDL (mmol/L), and TG (mmol/L) were measured using the Roche Chemistry Analyzer (cobas 8000 c702, Roche, Basel, Switzerland). All tests had low (<2%) intraday and interday coefficients of variation. Glucose levels from 77 samples were below the lower limit (i.e., 0.11 mmol/L) of the linear detection range and were therefore excluded from further analysis.

### Covariates

We collected information on maternal age, height, pre-pregnancy weight, parity, education level, monthly income, and active/passive smoking during pregnancy using a self-reported questionnaire at recruitment (around 16 weeks of gestation). Height and pre-pregnancy weight were used to calculate pre-pregnancy body mass index (BMI = weight [kg]/height [m]^2^), which was then categorized into underweight (BMI < 18.5 kg/m^2^), normal weight (BMI 18.5–23.9), and overweight/obesity (BMI ≥ 24) based on the Chinese standard (19). In addition, we obtained information on pregnancy complications [hypertensive disorder in pregnancy and gestational diabetes mellitus (GDM)] from the medical records after delivery. Hypertensive disorder in pregnancy was identified using medical record data based on the International Classification of Diseases, Tenth Revision (ICD-10) (O13–O16). The diagnosis of GDM was made using the International Association of Diabetes and Pregnancy Study Group criteria (20).

### Statistical Analysis

The levels of cord blood cardiometabolic factors (insulin, glucose, total cholesterol, triglyceride, HDL, and LDL) were log-transformed to address non-normality of their distributions. Since different units and scales were used for different cardiometabolic factors, we then converted the log-transformed variables to *z*-scores (using internal means and standard deviations) for ease of comparison of results.

We used multiple linear regression model to examine the association between fetal growth variables (*z*-scores and growth categories for weight, AC, and FL) at each period and the levels of each cord blood cardiometabolic factor, and the regression coefficient (*β*) and 95% CI were calculated. Covariates included in these models were maternal age, education level, monthly income, parity, pre-pregnancy BMI, active/passive smoking during pregnancy, hypertensive disorder in pregnancy, and GDM. In the models for fetal growth velocity in the early-third and late-third trimesters, we further adjusted for growth variables in previous periods. However, fetal size in early pregnancy was not included for adjustment because it was highly correlated to growth velocity in mid-pregnancy. In the models of fetal growth categories, the group of “normal” growth was used as a referent and compared with the groups of “slow” and “fast” growth. We first included fetal weight, AC, and FL separately in the models.

We conducted exploratory stratified analyses to investigate whether the associations between fetal growth *z*-scores and cord blood cardiometabolic factor levels differed by maternal characteristics such as pre-pregnancy BMI (underweight vs. normal weight and overweight/obesity), parity (nulliparous vs. multiparous), and GDM (yes vs. no), which have been shown to affect fetal growth or fetal metabolic status. Multiplicative interaction term was included in the multiple linear regression model to assess whether the interaction between fetal growth *z*-scores and these maternal and neonatal characteristics exists. In addition, we conducted sensitivity analysis for the associations between continuous fetal growth variables and cardiometabolic factors, by excluding preterm infants and women with hypertensive disorder in pregnancy.

All data were analyzed by SPSS 21.0, and the statistical significance was set as a two-tailed *P <*0.05.

## Results

Participant characteristics are presented in **Table 1**. The mean age of the participants was 29.5 years. Most of them (80%) were nulliparous. About one in four were underweight before pregnancy, whereas around one in 10 were overweight or obese. About 12% of these women had GDM, 3% had hypertensive disorders in pregnancy, and 29.3% were exposed to active or passive smoking during pregnancy. The mean birth weight of their infants was 3,203 g.

**Table 1:** Characteristics of the study population.

Variables	All	Boys	Girls
	*n* = 1458	*n* = 782	*n* = 676
Maternal characteristics			
Age (years), mean (SD)	29.5 (3.3)	29.5 (3.4)	29.5 (3.2)
Educational level (%)			
Middle school or below	89 (6.1)	44 (5.6)	45 (6.7)
Vocational or technical college	296 (20.3)	164 (21.0)	132 (19.5)
Undergraduate	844 (57.9)	455 (58.2)	389 (57.5)
Postgraduate	229 (15.7)	119 (15.2)	110 (16.3)
Monthly income (Yuan) (%)			
≤1,500	101 (6.9)	56 (7.2)	45 (6.7)
1,501–4,500	333 (22.8)	172 (22.0)	161 (23.8)
4,501–9,000	661 (45.3)	352 (45.0)	309 (45.7)
≥9,001	363 (24.9)	202 (25.8)	161 (23.8)
Pre-pregnancy BMI (kg/m^2^), mean (SD)	20.6 (2.7)	20.5 (2.7)	20.6 (2.7)
Underweight (%)	337 (23.1)	182 (23.3)	155 (22.9)
Normal weight (%)	968 (66.4)	519 (66.4)	449 (66.4)
Overweight/obesity (%)	153 (10.5)	81 (10.4)	72 (10.7)
Nulliparous (%)	1,167 (80.0)	625 (79.9)	542 (80.2)
Diabetes during pregnancy (%)	179 (12.3)	93 (11.9)	86 (12.7)
Hypertensive disorder in pregnancy (%)	44 (3.0)	20 (2.6)	24 (3.6)
Active/passive smoking (%)	427 (29.3)	224 (28.6)	203 (30.0)
Child’s characteristics			
Gestational age (weeks), median (25th–75th percentile)	39.0 (38.0–40.0)	39.0 (38.0–40.0)	39.0 (38.0–40.0)
Birth weight (grams), mean (SD)	3,203 (403)	3,238 (406)	3,162 (396)
Cord blood metabolic factors levels			
Insulin median (25th–75th), μIU/ml	7.42 (4.35–12.51)	7.00 (4.00–11.72)	8.07 (4.86–13.50)
Glucose median (25th–75th), mmol/L	4.96 (3.47–6.15)	5.10 (3.56–6.21)	4.79 (3.36–6.06)
Cholesterol median (25th–75th), mmol/L	1.68 (1.43–1.68)	1.61 (1.38–1.92)	1.72 (1.50–2.01)
Triglyceride median (25th–75th), mmol/L	0.33 (0.27–0.41)	0.34 (0.27–0.41)	0.33 (0.27–0.41)
LDL median (25th–75th), mmol/L	0.58 (0.44–0.73)	0.56 (0.42–0.71)	0.59 (0.46–0.75)
HDL median (25th–75th), mmol/L	0.87 (0.72–1.05)	0.84 (0.72–1.02)	0.91 (0.75–1.09)

SD, standard deviation.

### Fetal Weight Growth and Cord Blood Cardiometabolic Factors

**Figure 1:**
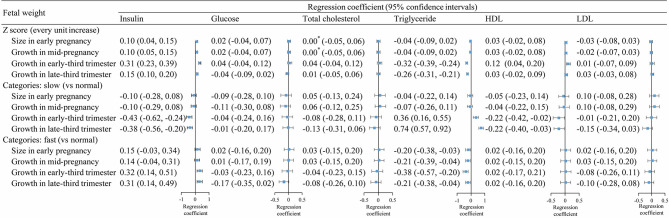
Associations between fetal weight growth in different periods and cord blood metabolic factors. *, >0 and <0.001.

Fetal weight *z*-scores at each gestational period were positively associated with cord blood insulin levels, with stronger associations observed in early- (*β*, 0.31; 95% CI, 0.23–0.39) and late-third trimesters (*β*, 0.15; 95% CI, 0.10 to 0.20) (**Figure 1**, upper section). Besides this, fetal weight *z*-scores in early- (*β*, −0.32; 95% CI, −0.39 to −0.24) and late-third (*β*, −0.26; 95% CI, −0.31 to −0.21) trimesters, but not early and mid-pregnancy, were negatively associated with cord blood TG levels. Fetal weight *z*-score in early-third trimester was also positively associated with HDL levels (*β*, 0.12; 95% CI, 0.04 to 0.20). No evident association was observed between fetal weight *z*-scores and glucose, total cholesterol, and LDL (**Figure 1**, upper section).

Results for fetal weight categories (slow, normal, or fast) were consistent with overall findings for fetal weight *z*-scores. Compared with normal-growth fetuses, slow-growth fetuses had lower levels of cord blood insulin in early- and late-third trimester, whereas fast-growth fetuses had higher insulin levels in early and late trimesters (**Figure 1**, middle and lower sections). In addition, slow-growth fetuses had higher levels of TG in early- and late-third trimester and lower levels of HDL at birth, whereas fast-growth fetuses had lower levels of TG in each gestational period.

### Fetal AC Growth and Cord Blood Cardiometabolic Factors

**Figure 2:**
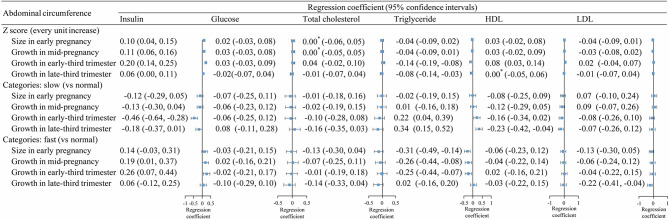
Association between fetal abdominal circumference growth in different periods and cord blood metabolic factors. *, >0 and <0.001.

The patterns of associations between fetal AC *z*-scores and cardiometabolic factors were similar to those for fetal weight growth, although the magnitude of the associations appeared to be smaller (**Figure 2**). The associations for fetal AC categories (slow, normal, or fast) were also similar to the overall results for fetal weight growth. However, the associations between AC categories in late-third trimester and insulin and TG were close to null, whereas the associations for fetal weight categories remained statistically significant.

### Fetal FL Growth and Cord Blood Cardiometabolic Factors

**Figure 3:**
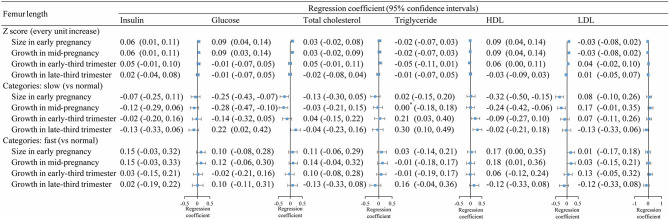
Association between fetal femur length growth in different periods and cord blood metabolic factors. *, >0 and <0.001.

Fetal FL *z*-scores in early and mid-pregnancy were associated with increased levels of cord blood insulin, glucose, and HDL (**Figure 3**, upper section). In line with these results, fetuses with slow FL growth in early and mid-pregnancy had lower levels of glucose and HDL at birth, while fetuses with fast FL growth had higher cord blood insulin and HDL levels (**Figure 3**, middle and lower sections). There was insufficient evidence for an association between FL continuous *z*-scores and cord blood TG levels. However, we found that babies with slow FL growth in early- and late-third trimesters had higher TG compared with those with normal FL growth.

### Stratified Analysis

Stratified analysis by GDM revealed negative associations of fetal weight and AC *z*-scores before 37 weeks of gestation with cord blood TG being stronger among women without diabetes than those with diabetes (*P*_interaction_, <0.05) (**Supplementary Tables 1**, **2**). No significant interaction was observed between FL and maternal diabetes status on cardiometabolic factors levels (**Supplementary Table 3**).

There was no evident interaction between fetal growth variables and maternal pre-pregnancy BMI (**Supplementary Tables 4**–**6**) and parity (**Supplementary Tables 7**–**9**) for any of the cardiometabolic factors.

### Sensitivity Analyses

In sensitivity analyses, the results were similar to the overall analysis when we excluded women with hypertensive disorder in pregnancy (*n* = 44) (**Supplementary Table 10**) or when we excluded preterm infants (*n* = 61) (**Supplementary Table 11**).

## Discussion

### Main Findings

We found that fetal weight at each period was positively associated with cord blood insulin, with stronger associations observed in early- and late-third trimesters than early and mid-pregnancy. Fetal weight in early- and late-third trimesters was also negatively associated with TG and positively associated with HDL. Associations for fetal AC were similar to those for fetal weight. These results suggest that the third trimester could be the important period for fetal insulin and lipid metabolism. On the other hand, fetal FL in the early and mid-pregnancy was positively associated with cord blood insulin, glucose, and HDL, indicating that the first half of pregnancy might be a crucial stage for the interplay between fetal skeletal growth and these metabolic factors. To our knowledge, this is the first study to investigate the associations between fetal growth in different gestational periods and cord blood cardiometabolic factors.

### Interpretation of the Results

Our observation that fetal weight and AC were positively associated with cord blood insulin is consistent with previous studies. Ong et al. found that cord blood insulin was positively associated with neonatal birth weight (21). Similarly, SGA neonates had hypoinsulinemia in cord blood at birth compared with AGA neonates (22), whereas insulin levels in LGA infants were significantly higher (21, 23). In addition, a previous study found gestational weight gain rates during the first and second trimesters, but not the third trimester, to be positively associated with cord blood insulin levels (24). In line with this study, we observed that fetal weight at early and mid-pregnancy was associated with insulin levels at birth; however, in contrast to this previous study (24), we found that fetal weight in the third trimester was also associated with cord blood insulin. Discrepancies in the results between the two studies might be related to the difference in exposure definition (e.g., self-reported gestational weight gain vs. ultrasound-based fetal growth), and thus, the results might be not directly comparable.

Our study revealed the association of fetal weight and AC with cord blood insulin to be stronger in late pregnancy than in early pregnancy. Insulin is an important hormone to promote fetal growth. It can not only stimulate fetal insulin sensitive cells to promote tissue utilization of glucose but also promote the storage of glycogen and fat, thereby promoting the growth of the fetus (25). Since fetal growth is fastest in late pregnancy (26), we speculate that fetuses in late pregnancy might be more sensitive to the growth-promoting effect of insulin than fetuses in early pregnancy. On the other hand, fetal growth may affect the levels of circulating insulin. It has been shown that fetal growth restriction can affect the maturation of fetal islet β cells, lead to the inhibition of fetal islet β-cell proliferation, reduce insulin secretion, hinder the activation of amino acid transport system, inhibit protein and fat synthesis, promote lipolysis, and inhibit fetal growth and development (27). These responses enable the fetus to adapt to the adverse intrauterine environment and redistribute nutrients, which ensures the growth and development of the main organs (e.g., brain) of the fetus but results in decreased fetal growth rate (28).

In addition, we found that in the second and third trimesters of pregnancy, fetal weight and AC were negatively associated with cord blood TG. This finding is consistent with previous studies which found that fetuses with intrauterine growth restriction had higher cord blood TG compared with normal-growth fetus (29, 30). Another study showed that AC was negatively associated with cord blood TG levels among growth-restricted fetuses (31). We also found that fetal weight and AC in late pregnancy were positively correlated with cord blood HDL. Interestingly, it has been shown that AC at birth was negatively associated with blood lipid levels in adulthood (32). Taken together, these findings suggest that abnormal fetal growth in late pregnancy might be critical for long-term lipid profile. A possible explanation for these findings is that decelerated fetal growth (especially for AC) may represent insufficient growth of the liver, which could result in dysregulation of lipid metabolism (32). However, the mechanisms for this association need to be studied in future studies.

The relationships between fetal FL or birth length and cord blood cardiometabolic factors have been rarely investigated previously. In the present study, we observed that femur growth in early and mid-pregnancy was positively associated with cord blood insulin and glucose. Fetal insulin, in addition to being a growth hormone, is also an osteogenic hormone, which can stimulate fetal bone mineralization and cartilage synthesis (33) and regulate fetal bone growth (34). Similarly, glucose is essential for fetal osteogenesis and cartilage formation. In the skeletal system, glucose is an important energy source for the development, growth, and maintenance of bone and articular cartilage (35). Therefore, insulin and glucose are two important nutrients for fetal FL growth in the early stage of pregnancy. We also found fetal FL in early and mid-pregnancy to be positively associated with cord blood HDL. The biological explanation for this association warrants further investigation.

In stratified analysis, we found that GDM modified the associations between fetal growth (weight and AC) and cord blood TG. For example, for pregnant women with diabetes, fetal weight growth before 28 weeks of gestation was positively associated with cord blood TG. By contrast, among women without diabetes, fetal weight growth in the same period was negatively associated with TG. This divergence of association was less pronounced after 28 weeks of gestation. We speculate that it could be related to the metabolic dysfunction induced by GDM. Fetal hyperinsulinemia, resulting from maternal GDM, can promote the synthesis of fat and leads to fetal overgrowth. This effect might be attenuated after 28 weeks when the dietary interventions are usually given to GDM patients for optimal glycemic control to improve pregnancy outcomes (36).

### Implications

In this study, we found a positive association between fetal weight and the levels of insulin and high-density lipoprotein in cord blood metabolic factors in all gestational periods, especially during the third trimester. Specifically, slow-growth fetuses in the third trimester had an adverse lipid profile (i.e., high TG and low HDL) at birth, whereas fast-growth fetuses in the third trimester had higher insulin insensitivity (reflected by higher cord blood insulin level). Since adverse cardiometabolic profile at birth may be related to long-term cardiometabolic diseases, efforts should be made to ensure an optimal fetal growth during late pregnancy to improve the short- and long-term cardiometabolic health. However, future studies are still needed to validate our findings.

### Strengths and Limitations

Based on a series of ultrasound measurements, we were able to assess longitudinal fetal growth patterns and their associations with cord blood cardiometabolic factors, which have not been reported previously. The prospective cohort design of the current study could reduce the recall bias for data collection. Compared with previous studies on birth weight and cord blood cardiometabolic factors (4, 9, 37), our study had relatively larger sample size and thus increased statistical power. A limitation of our study was that we could only measure metabolic factors in umbilical cord blood at birth. Information about the fetal metabolic status before birth would be useful, which allows the assessment for the dynamic changes of fetal metabolic status. However, it is impractical to collect cord blood samples before birth for normal pregnancies. Another limitation is that only a limited number of cardiometabolic factors were measured and data on adipokines (e.g., leptin and adiponectin), which may also play a role in fetal growth, were unavailable.

## Conclusions

This study shows that fetal growth in different gestational periods was associated with cord blood cardiometabolic factors. These associations were more pronounced for fetal weight and AC growth after 28 weeks than those before 28 weeks, whereas associations for FL growth appeared to stronger before 28 weeks. Our findings suggest that fetal growth and cardiometabolic factors might interplay since early pregnancy. Further studies are needed to elucidate the mechanisms underlying these associations.

## Data Availability Statement

The original contributions presented in the study are included in the article/**Supplementary Material**. Further inquiries can be directed to the corresponding authors.

## Ethics Statement

The studies involving human participants were reviewed and approved by the Ethics Committee of Guangzhou Women and Children’s Medical Center. The patients/participants provided their written informed consent to participate in this study.

## Author Contributions

J-RH and XQ conceptualized and designed the study, contributed to the data analysis, interpreted the data, and reviewed and revised the manuscript. J-SH, Q-ZC, S-YZ, J-YZ, and C-PZ analyzed and interpreted the data and draft and revised the manuscript. RR contributed to the data analysis and reviewed and revised the manuscript. Y-ML, Y-SK, and J-HL contributed to the data collection and reviewed and revised the manuscript. All authors approved the final manuscript as submitted and agree to be accountable for all aspects of the work.

## Funding

The present study was supported by the grants from Ministry of Science and Technology of People's Republic of China (2019YFC0121905) and National Natural Science Foundation of China (grant number, 81703244 and 81673181).

## Conflict of Interest

The authors declare that the research was conducted in the absence of any commercial or financial relationships that could be construed as a potential conflict of interest.

## Publisher’s Note

All claims expressed in this article are solely those of the authors and do not necessarily represent those of their affiliated organizations, or those of the publisher, the editors and the reviewers. Any product that may be evaluated in this article, or claim that may be made by its manufacturer, is not guaranteed or endorsed by the publisher.
