# Supplementary material for: Associations of Longitudinal Fetal Growth Patterns With Cardiometabolic Factors at Birth
Source: Front Endocrinol (Lausanne). 2021 Dec 9;12:771193. doi: 10.3389/fendo.2021.771193 (PMC8696025; doi:10.3389/fendo.2021.771193)
Supplement: Supplementary file 1 [file Table_1.pdf]

Supplementary table 1. Stratified associations between fetal weight at different stages and cord blood metabolic factors by maternal diabetes status

Periods	Stratum	Regression coefficients (95% confidence intervals)					
		Insulin	Glucose	Total cholesterol	Triglyceride	HDL	LDL
<22 weeks	No Diabetes	0.10 (0.04, 0.15)	0.03 (-0.03, 0.08)	0.01 (-0.05, 0.06)	-0.06 (-0.12, <-0.001^)	0.05 (-0.01, 0.10)	-0.03 (-0.09, 0.03)
	Diabetes	0.08 (-0.05, 0.21)	-0.02 (-0.17, 0.14)	0.02 (-0.17, 0.13)	0.11 (-0.03, 0.26)	-0.10 (-0.24, 0.04)	-0.02 (-0.13, 0.17)
	$P_{interaction}$	0.82	0.45	0.88	0.03	0.11	0.44
22-27 weeks	No Diabetes	0.10 (0.05, 0.16)	0.03 (-0.03, 0.08)	0.01 (-0.05, 0.06)	-0.06 (-0.12, -0.01)	0.05 (-0.01, 0.10)	-0.03 (-0.09, 0.03)
	Diabetes	0.08 (-0.05, 0.21)	-0.02 (-0.17, 0.13)	-0.02 (-0.17, 0.12)	0.11 (-0.03, 0.26)	-0.10 (-0.24, 0.04)	0.02 (-0.13, 0.17)
	$P_{interaction}$	0.79	0.43	0.86	0.03	0.10	0.44
28-36 weeks	No Diabetes	0.30 (0.21, 0.38)	0.04 (-0.04, 0.13)	0.04 (-0.05, 0.12)	-0.33 (-0.41, -0.25)	0.13 (-0.05, 0.21)	0.00 (-0.08, 0.09)
	Diabetes	0.37 (0.15, 0.59)	-0.04 (-0.32, 0.23)	0.02 (-0.24, 0.28)	-0.20 (-0.44, 0.04)	0.03 (-0.22, 0.27)	0.02 (-0.24, 0.28)
	$P_{interaction}$	0.49	0.48	0.96	0.01	0.09	0.37
≥37 weeks	No Diabetes	0.15 (0.09, 0.20)	-0.02 (-0.08, 0.04)	0.01 (-0.05, 0.07)	-0.27 (-0.33, -0.22)	0.04 (-0.02, 0.10)	0.02 (-0.04, 0.08)
	Diabetes	0.20 (0.05, 0.36)	-0.19 (-0.38, <0.01)	-0.05 (-0.23, 0.13)	-0.23 (-0.40, -0.07)	-0.06 (-0.23, 0.11)	0.07 (-0.12, 0.25)
	$P_{interaction}$	0.27	0.10	0.70	0.25	0.23	0.34

HDL, high-density lipoprotein cholesterol; LDL, low-density lipoprotein cholesterol.

Supplementary table 2. Stratified associations between fetal abdominal circumference at different growth stages and cord blood metabolic factors by maternal diabetes status

Periods	Stratum	Regression coefficients (95% confidence intervals)					
		Insulin	Glucose	Total cholesterol	Triglyceride	HDL	LDL
<22 weeks	No Diabetes	0.09 (0.04, 0.15)	0.02 (-0.03, 0.08)	<-0.001^ (-0.06, 0.05)	-0.06 (-0.11, <-0.001^)	0.04 (-0.01, 0.10)	-0.04 (-0.10, 0.01)
	Diabetes	0.09 (-0.06, 0.24)	0.07 (-0.11, 0.25)	-0.04 (-0.21, 0.13)	0.14 (-0.02, 0.30)	-0.11 (-0.27, 0.05)	-0.01 (-0.18, 0.17)
	$P_{interaction}$	0.94	0.92	0.89	0.04	0.20	0.66
22-27 weeks	No Diabetes	0.11 (0.05, 0.16)	0.03 (-0.03, 0.08)	<0.01 (-0.05, 0.06)	-0.06 (-0.12, -0.01)	0.05 (-0.01, 0.10)	-0.04 (-0.09, 0.02)
	Diabetes	0.11 (-0.04, 0.26)	0.07 (-0.11, 0.24)	-0.04 (-0.21, 0.13)	0.14 (-0.01, 0.30)	-0.12 (-0.27, 0.04)	<0.01 (-0.17, 0.17)
	$P_{interaction}$	0.90	0.92	0.88	0.02	0.15	0.59
28-36 weeks	No Diabetes	0.20 (0.13, 0.26)	0.03 (-0.03, 0.10)	0.04 (-0.02, 0.10)	-0.15 (-0.22, -0.09)	0.10 (0.03, 0.16)	0.01 (-0.05, 0.07)
	Diabetes	0.19 (0.03, 0.34)	-0.05 (-0.24, 0.14)	0.04 (-0.13, 0.22)	-0.04 (-0.20, 0.13)	0.03 (-0.14, 0.20)	0.01 (-0.17, 0.19)
	$P_{interaction}$	0.64	0.86	0.10	0.02	0.16	0.63
≥37 weeks	No Diabetes	0.06 (0.01, 0.12)	-0.02 (-0.08, 0.04)	-0.02 (-0.08, 0.04)	-0.09 (-0.15, -0.03)	0.01 (-0.05, 0.06)	-0.02 (-0.08, 0.04)
	Diabetes	-0.03 (-0.21, 0.16)	-0.04 (-0.27, 0.19)	0.01 (-0.20, 0.22)	0.04 (-0.16, 0.24)	-0.03 (-0.23, 0.18)	0.07 (-0.15, 0.28)
	$P_{interaction}$	0.63	0.97	0.86	0.05	0.34	0.26

^, >-0.01 and <-0.001

HDL, high-density lipoprotein cholesterol; LDL, low-density lipoprotein cholesterol.

Supplementary table 3. Stratified associations between fetal femur length at different growth stages and cord blood metabolic factors by maternal diabetes status

Periods	Stratum	Regression coefficients (95% confidence intervals)					
		Insulin	Glucose	Total cholesterol	Triglyceride	HDL	LDL
<22 weeks	No Diabetes	0.05 (<-0.001^, 0.11)	0.10 (0.04,0.15)	0.04 (-0.02, 0.09)	-0.03 (-0.09, 0.02)	0.10 (0.04, 0.15)	-0.03 (-0.08, 0.03)
	Diabetes	0.09 (-0.06, 0.23)	<-0.001^ (-0.18, 0.17)	-0.01 (-0.17, 0.15)	0.06 (-0.10, 0.21)	0.02 (-0.13, 0.17)	-0.08 (-0.24, 0.08)
	$P_{interaction}$	0.65	0.34	0.64	0.36	0.39	0.63
22-27 weeks	No Diabetes	0.06(<-0.001^, 0.11)	0.10 (0.04, 0.15)	0.04 (-0.02, 0.09)	-0.03 (-0.08, 0.03)	0.10 (0.04, 0.15)	-0.02 (-0.08, 0.03)
	Diabetes	0.10 (-0.04, 0.24)	-0.01 (-0.19, 0.16)	0.01 (-0.15, 0.17)	0.06 (-0.09, 0.21)	0.04 (-0.12, 0.19)	-0.06 (-0.22, 0.11)
	$P_{interaction}$	0.54	0.31	0.90	0.35	0.51	0.86
28-36 weeks	No Diabetes	0.05 (-0.02, 0.11)	<0.01(-0.06, 0.07)	0.04 (-0.03, 0.10)	-0.04 (-0.11, 0.02)	0.05 (-0.01, 0.11)	0.03 (-0.03, 0.09)
	Diabetes	0.03 (-0.12, 0.19)	<-0.001^ (-0.28,0.08)	0.11 (-0.07, 0.29)	-0.09 (-0.26, 0.07)	0.07 (-0.10, 0.24)	0.12 (-0.06, 0.29)
	$P_{interaction}$	0.84	0.18	0.48	0.91	0.91	0.50
≥37 weeks	No Diabetes	0.03 (-0.03, 0.09)	<0.01(-0.06, 0.07)	-0.02 (-0.08, 0.05)	-0.01 (-0.07, 0.06)	-0.03 (-0.09, 0.03)	0.02 (-0.05, 0.08)
	Diabetes	-0.08 (-0.26, 0.10)	-0.11 (-0.32, 0.10)	-0.08 (-0.28, 0.12)	<-0.001^ (-0.19, 0.19)	-0.05 (-0.24, 0.14)	-0.02 (-0.22, 0.18)
	$P_{interaction}$	0.33	0.10	0.93	0.80	0.85	0.80

^, >-0.01 and <-0.001

HDL, high-density lipoprotein cholesterol; LDL, low-density lipoprotein cholesterol.

Supplementary table 4. Stratified associations between fetal weight at different stages and cord blood metabolic factors by maternal pre-pregnancy BMI

Periods	Stratum	Regression coefficients (95% confidence intervals)					
		Insulin	Glucose	Total cholesterol	Triglyceride	HDL	LDL
<22 weeks	Underweight	0.04 (-0.08, 0.16)	0.11 (0.01, 0.21)	0.02 (-0.09, 0.13)	0.01 (-0.11, 0.13)	0.02 (-0.10, 0.14)	<-0.001^ (-0.11, 0.10)
	Normal	0.11 (0.05, 0.17)	0.01 (-0.06, 0.08)	<-0.001^ (-0.07, 0.06)	-0.05 (-0.11, 0.02)	0.04 (-0.03, 0.10)	-0.03 (-0.09, 0.04)
	Overweight/obesity	0.11 (0.04, 0.26)	-0.06 (-0.23, 0.12)	0.023 (-0.15, 0.19)	0.02 (-0.16, 0.20)	-0.01 (-0.15, 0.14)	<0.01 (-0.19, 0.19)
	$P_{interaction}$	0.48	0.10	0.68	0.77	0.65	0.42
22-27 weeks	Underweight	0.04 (-0.08, 0.16)	0.11 (0.01, 0.21)	0.02 (-0.09, 0.13)	0.01 (-0.11, 0.13)	0.02 (-0.10, 0.13)	<-0.001^ (-0.11, 0.11)
	Normal	0.11 (0.05, 0.17)	0.01 (-0.06, 0.08)	<0.001^ (-0.06, 0.06)	-0.05 (-0.11, 0.02)	0.04 (-0.03, 0.10)	-0.03 (-0.09, 0.04)
	Overweight/obesity	0.12 (-0.04, 0.27)	-0.06 (-0.24, 0.11)	0.03 (-0.15, 0.19)	0.03 (-0.17, 0.20)	-0.01 (-0.16, 0.14)	<0.01 (-0.19, 0.19)
	$P_{interaction}$	0.48	0.11	0.68	0.77	0.64	0.42
28-36 weeks	Underweight	0.42 (0.26, 0.58)	0.02 (-0.13, 0.16)	0.11 (-0.04, 0.26)	-0.39 (-0.55, -0.23)	0.22 (0.06, 0.38)	0.03 (-0.12, 0.18)
	Normal	0.27 (0.17, 0.36)	0.02 (-0.08, 0.13)	0.02 (-0.08, 0.12)	-0.30 (-0.39, 0.20)	0.08 (-0.02, 0.18)	0.02 (-0.08, 0.12)
	Overweight/obesity	0.33 (0.12, 0.54)	0.12 (-0.14, 0.37)	-0.05 (-0.29, 0.20)	-0.31 (-0.55, -0.07)	0.11 (-0.10, 0.32)	-0.12 (-0.40, 0.15)
	$P_{interaction}$	0.89	0.58	0.63	0.50	0.30	0.94
≥37 weeks	Underweight	0.14 (0.03, 0.26)	-0.01 (-0.11, 0.09)	-0.01 (-0.12, 0.10)	-0.39 (-0.50, -0.28)	0.11 (-0.01, 0.23)	-0.04 (-0.15, 0.07)
	Normal	0.15 (0.09, 0.22)	-0.01 (-0.08, 0.06)	<0.01 (-0.07, 0.07)	-0.24 (-0.30, -0.17)	0.01 (-0.06, 0.08)	0.03 (-0.03, 0.10)
	Overweight/obesity	0.09 (-0.06, 0.24)	-0.26 (-0.44, -0.09)	0.02 (-0.15, 0.19)	-0.14 (-0.31, 0.02)	-0.02 (-0.17, 0.13)	0.12 (-0.07, 0.31)
	$P_{interaction}$	0.47	0.55	0.80	<0.01	0.22	0.25

*, >0 and <0.001

^, >-0.01 and <-0.001

HDL, high-density lipoprotein cholesterol; LDL, low-density lipoprotein cholesterol.

Supplementary table 5. Stratified associations between fetal abdominal circumference at different growth stages and cord blood metabolic factors by maternal pre pregnancy BMI

Periods	Stratum	Regression coefficients (95% confidence intervals)					
		Insulin	Glucose	Total cholesterol	Triglyceride	HDL	LDL
<22 weeks	Underweight	0.06 (-0.05, 0.17)	0.08 (-0.01, 0.18)	0.03(-0.07, 0.14)	-0.04 (-0.15, 0.08)	0.06 (-0.05, 0.17)	-0.02 (-0.12, 0.08)
	Normal	0.10 (0.03, 0.16)	0.01 (-0.06, 0.08)	-0.01 (-0.08, 0.05)	-0.04 (-0.10, 0.02)	0.02 (-0.04, 0.09)	-0.04 (-0.10, 0.03)
	Overweight/obesity	0.15 (<0.01, 0.30)	0.02 (-0.17, 0.20)	<0.01 (-0.17, 0.17)	0.04 (-0.14, 0.21)	0.02 (-0.13, 0.17)	-0.05 (-0.24, 0.15)
	$P_{interaction}$	0.45	0.28	0.57	0.55	0.31	0.97
22-27 weeks	Underweight	0.08 (-0.04, 0.19)	0.09 (-0.01, 0.18)	0.04 (-0.06, 0.14)	-0.04 (-0.15, 0.07)	0.06 (-0.05, 0.17)	-0.01 (-0.12, 0.09)
	Normal	0.11 (0.05, 0.17)	0.02 (-0.05, 0.09)	-0.01 (-0.07, 0.06)	-0.04 (-0.11, 0.02)	0.03 (-0.04, 0.09)	-0.03 (-0.10, 0.03)
	Overweight/obesity	0.17 (0.02, 0.32)	<0.01(0.18, 0.18)	0.01 (-0.17, 0.18)	0.02 (-0.16, 0.20)	0.03 (-0.13, 0.18)	-0.04 (-0.23, 0.16)
	$P_{interaction}$	0.42	0.30	0.59	0.60	0.28	0.93
28-36 weeks	Underweight	0.30 (0.18, 0.42)	0.01 (-0.10, 0.11)	0.06 (-0.05, 0.17)	-0.19 (-0.31, -0.07)	0.12 (<-0.001^, 0.24)	0.03 (-0.09, 0.14)
	Normal	0.16 (0.09, 0.24)	0.03 (-0.05, 0.10)	0.03 (-0.04, 0.10)	-0.12 (-0.19, 0.05)	0.07 (<-0.001^, 0.14)	0.01 (-0.06, 0.09)
	Overweight/obesity	0.18 (0.02, 0.34)	0.09 (-0.11, 0.30)	0.05 (-0.14, 0.23)	-0.16 (-0.34, 0.02)	0.09 (-0.07, 0.25)	-0.02 (-0.22, 0.19)
	$P_{interaction}$	0.60	0.54	0.69	0.70	0.38	0.91
≥37 weeks	Underweight	0.12 (<0.01, 0.23)	0.02 (-0.08, 0.12)	-0.02 (-0.13, 0.08)	-0.16 (-0.27, -0.04)	0.07 (-0.05, 0.19)	-0.06 (-0.17, 0.04)
	Normal	0.04 (-0.03, 0.12)	-0.03 (-0.10, 0.05)	-0.02 (-0.10, 0.05)	-0.08 (-0.15, <-0.001^)	-0.03 (-0.10, 0.05)	<-0.001^ (-0.08, 0.07)
	Overweight/obesity	-0.02 (-0.15, 0.12)	-0.05 (-0.22, 0.11)	0.01 (-0.15, 0.16)	0.05 (-0.10, 0.21)	-0.02 (-0.15, 0.11)	0.03 (-0.14, 0.21)
	$P_{interaction}$	0.21	0.73	0.32	0.06	0.66	0.11

^, >-0.01 and <-0.001

HDL, high-density lipoprotein cholesterol; LDL, low-density lipoprotein cholesterol.

Supplementary table 6. Stratified associations between fetal femur length at different growth stages and cord blood metabolic factors by maternal pre-pregnancy BMI

Periods	Stratum	Regression coefficients (95% confidence intervals)					
		Insulin	Glucose	Total cholesterol	Triglyceride	HDL	LDL
<22 weeks	Underweight	0.08 (-0.03, 0.19)	0.05 (-0.05, 0.14)	0.06 (-0.04, 0.16)	-0.05 (-0.16, 0.05)	0.13 (0.03, 0.24)	-0.01 (-0.11, 0.08)
	Normal	0.04 (-0.02, 0.11)	0.12 (0.06, 0.19)	0.03 (-0.04, 0.09)	-0.01 (-0.07, 0.05)	0.08 (0.02, 0.15)	-0.03 (-0.09, 0.04)
	Overweight/obesity	0.09 (-0.06, 0.25)	-0.01 (-0.20, 0.17)	0.05 (-0.12, 0.23)	0.03 (-0.15, 0.21)	0.08 (-0.07, 0.23)	-0.04 (-0.24, 0.15)
	$P_{interaction}$	0.77	0.43	0.99	0.33	0.66	0.82
22-27 weeks	Underweight	0.08 (-0.03, 0.19)	0.04 (-0.05, 0.13)	0.07 (-0.03, 0.17)	-0.05 (-0.16, 0.05)	0.13 (0.03, 0.24)	<0.01 (-0.10, 0.10)
	Normal	0.05 (-0.02, 0.11)	0.13 (0.06, 0.20)	0.03 (-0.04, 0.09)	-0.01 (-0.07, 0.06)	0.08 (0.01, 0.14)	-0.03 (-0.09, 0.04)
	Overweight/obesity	0.10 (-0.06, 0.25)	-0.04 (-0.22, 0.15)	0.07 (-0.10, 0.25)	0.02 (-0.16, 0.20)	0.10 (-0.05, 0.25)	-0.03 (-0.22, 0.17)
	$P_{interaction}$	0.77	0.38	0.91	0.35	0.74	0.85
28-36 weeks	Underweight	0.08 (-0.05, 0.21)	0.02 (-0.09, 0.13)	0.07 (-0.05, 0.18)	-0.10 (-0.22, 0.03)	0.07 (-0.05, 0.20)	0.06 (-0.06, 0.17)
	Normal	0.03 (-0.04, 0.10)	-0.02 (-0.09, 0.06)	0.02 (-0.06, 0.09)	-0.03 (-0.10, 0.04)	0.02 (-0.05, 0.09)	0.03 (-0.05, 0.10)
	Overweight/obesity	0.09 (-0.08, 0.26)	-0.07 (-0.27, 0.13)	0.22 (0.03, 0.41)	-0.06 (-0.25, 0.13)	0.23 (0.07, 0.39)	0.13 (-0.08, 0.34)
	$P_{interaction}$	0.84	0.19	0.08	0.98	0.08	0.35
≥37 weeks	Underweight	0.11 (-0.02, 0.23)	0.03 (-0.08, 0.14)	-0.01 (-0.12, 0.10)	-0.06 (-0.18, 0.07)	0.05 (-0.08, 0.17)	-0.07 (-0.18, 0.05)
	Normal	-0.03 (-0.10, 0.04)	-0.02 (-0.10, 0.06)	-0.03 (-0.11, 0.04)	0.02 (-0.06, 0.09)	-0.06 (-0.13, 0.01)	0.03 (-0.04, 0.10)
	Overweight/obesity	0.15 (-0.04, 0.34)	<-0.001^ (-0.24, 0.23)	0.01 (-0.21, 0.22)	-0.04 (-0.26, 0.18)	-0.07 (-0.26, 0.11)	0.14 (-0.10, 0.38)
	$P_{interaction}$	0.33	0.16	0.26	0.85	0.82	0.03

*, >0 and <0.001

^, >-0.01 and <-0.001

HDL, high-density lipoprotein cholesterol; LDL, low-density lipoprotein cholesterol.

Supplementary table 7. Stratified associations between fetal weight at different stages and cord blood metabolic factors by maternal parity

Periods	Stratum	Regression coefficients (95% confidence intervals)					
		Insulin	Glucose	Total cholesterol	Triglyceride	HDL	LDL
<22 weeks	Primipara	0.09 (0.03, 0.14)	0.01 (-0.04, 0.07)	0.01 (-0.05, 0.06)	-0.04 (-0.10, 0.02)	0.05 (-0.01, 0.10)	-0.03 (-0.08, 0.03)
	Multipara	0.14 (0.03, 0.24)	0.03 (-0.12, 0.19)	-0.03 (-0.15, 0.09)	0.01 (-0.10, 0.13)	-0.06 (-0.18, 0.06)	-0.03 (-0.15, 0.09)
	$P_{interaction}$	0.52	0.74	0.86	0.62	0.21	0.79
22-27 weeks	Primipara	0.09 (0.03, 0.15)	0.01 (-0.04, 0.07)	0.01 (-0.05, 0.06)	-0.04 (-0.10, 0.02)	0.04 (-0.01, 0.10)	-0.03 (-0.08, 0.03)
	Multipara	0.14 (0.03, 0.24)	0.04 (-0.12, 0.19)	-0.03 (-0.15, 0.10)	0.01 (-0.10, 0.13)	-0.06 (-0.18, 0.06)	-0.03 (-0.15, 0.10)
	$P_{interaction}$	0.54	0.70	0.89	0.60	0.22	0.76
28-36 weeks	primipara	0.30 (0.21, 0.39)	0.04 (-0.05, 0.12)	0.01 (-0.08, 0.10)	-0.29 (-0.38, 0.21)	0.10 (0.01, 0.19)	-0.02 (-0.11, 0.06)
	Multipara	0.36 (0.21, -0.51)	0.01 (-0.22, 0.23)	0.15 (-0.03, 0.34)	-0.43 (-0.59, 0.27)	-0.19 (0.01, 0.36)	0.14 (-0.04, 0.32)
	$P_{interaction}$	0.40	0.80	0.42	0.78	0.77	0.24
≥37 weeks	Primipara	0.15 (0.09, 0.21)	-0.06 (-0.12, <-0.001^)	0.01 (-0.05, 0.07)	-0.30 (-0.36, -0.24)	0.04 (-0.02, 0.10)	0.03 (-0.03, 0.09)
	Multipara	0.11 (<0.01, 0.22)	0.09 (-0.08, 0.25)	-0.02 (-0.15, 0.11)	-0.10 (-0.21, 0.02)	-0.02 (-0.15, 0.11)	-0.01 (-0.14, 0.12)
	$P_{interaction}$	0.94	0.30	0.85	0.10	0.75	0.96

^, >-0.01 and <-0.001

HDL, high-density lipoprotein cholesterol; LDL, low-density lipoprotein cholesterol.

Supplementary table 8. Stratified associations between fetal abdominal circumference at different growth stages and cord blood metabolic factors by maternal parity

Periods	Stratum	Regression coefficients (95% confidence intervals)					
		Insulin	Glucose	Total cholesterol	Triglyceride	HDL	LDL
<22 weeks	Primipara	0.09 (0.03, 0.14)	0.03 (-0.03, 0.09)	-0.01 (-0.06, 0.05)	-0.04 (-0.09, 0.02)	0.04 (-0.02, 0.10)	-0.04 (-0.10， 0.02)
	Multipara	0.12 (0.02, 0.23)	-0.01 (-0.16, 0.14)	-0.01 (-0.13, 0.11)	-0.03 (-0.14, 0.08)	-0.02 (-0.14, 0.10)	-0.03 (-0.15, 0.08)
	$P_{interaction}$	0.58	0.67	0.90	0.94	0.48	0.72
22-27 weeks	Primipara	0.10 (0.04, 0.16)	0.03 (-0.02, 0.09)	<-0.001^ (-0.06, 0.05)	-0.04 (-0.10, 0.02)	0.04 (-0.02, 0.10)	-0.04 (-0.10, 0.02)
	Multipara	0.14 (0.03, 0.24)	<-0.001^ (-0.16, 0.15)	<0.01 (-0.12, 0.12)	-0.03 (-0.14, 0.08)	-0.01 (-0.13, 0.11)	-0.02 (-0.14, 0.10)
	$P_{interaction}$	0.63	0.70	0.76	0.98	0.57	0.63
28-36 weeks	primipara	0.20 (0.13, 0.26)	0.04 (-0.03, 0.10)	0.01 (-0.05, 0.07)	-0.13 (-0.20, 0.07)	0.06 (<-0.001^, 0.13)	-0.01 (-0.08, 0.05)
	Multipara	0.22 (0.10, 0.34)	-0.03 (-0.22, 0.15)	0.18 (-0.04, 0.32)	-0.15 (-0.28, 0.02)	0.17 (0.03, 0.31)	0.16 (0.02, 0.30)
	$P_{interaction}$	0.55	0.47	0.05	0.96	0.37	0.05
≥37 weeks	Primipara	0.07 (0.01, 0.13)	-0.02 (-0.08, 0.04)	-0.01 (-0.07, 0.06)	-0.11 (-0.18, -0.05)	0.02 (-0.05, 0.08)	<-0.001^ (-0.06, 0.06)
	Multipara	-0.02 (-0.14, 0.10)	0.02 (-0.16, 0.20)	-0.07 (-0.21, 0.07)	0.07 (-0.06, 020)	-0.08 (-0.21, 0.06)	-0.08 (-0.22, 0.05)
	$P_{interaction}$	0.43	0.82	0.92	0.02	0.49	0.74

^, >-0.01 and <-0.001

HDL, high-density lipoprotein cholesterol; LDL, low-density lipoprotein cholesterol.

Supplementary table 9. Stratified associations between fetal femur length at different growth stages and cord blood metabolic factors by maternal parity

Periods	Stratum	Regression coefficients (95% confidence intervals)					
		Insulin	Glucose	Total cholesterol	Triglyceride	HDL	LDL
<22 weeks	Primipara	0.05 (-0.01, 0.10)	0.08 (0.03, 0.14)	0.04 (-0.02, 0.10)	0.05 (-0.01, 0.10)	0.10 (0.04, 0.16)	-0.02 (-0.08, 0.03)
	Multipara	0.10 (-0.01, 0.21)	0.11 (-0.06, 0.27)	<0.001^ (-0.13, 0.12)	0.10 (-0.01, 0.21)	0.03 (-0.09, 0.16)	-0.05 (-0.17, 0.08)
	$P_{interaction}$	0.33	0.45	0.50	0.74	0.37	0.61
22-27 weeks	Primipara	0.05 (-0.01, 0.11)	0.08 (0.02, 0.13)	0.04 (-0.02, 0.10)	-0.02 (-0.07, 0.04)	0.10 (0.04, 0.16)	-0.02 (-0.08, 0.04)
	Multipara	0.10 (-0.01, 0.21)	0.11 (-0.06, 0.27)	<0.01(-0.13, 0.13)	-0.04 (-0.16, 0.08)	0.04 (-0.09, 0.17)	-0.04 (-0.17, 0.09)
	$P_{interaction}$	0.35	0.44	0.57	0.68	0.45	0.66
28-36 weeks	primipara	0.04 (-0.03, 0.11)	-0.01 (-0.07, 0.06)	0.05 (-0.02, 0.11)	-0.04 (-0.10, 0.03)	0.05 (-0.01, 0.12)	0.03 (-0.03, 0.10)
	Multipara	0.06 (-0.06, 0.18)	-0.04 (-0.22, 0.14)	0.06 (-0.08, 0.20)	-0.12 (-0.25, 0.01)	0.08 (-0.06, 0.22)	0.10 (-0.04, 0.23)
	$P_{interaction}$	0.39	0.94	0.96	0.25	0.96	0.61
≥37 weeks	Primipara	0.02 (-0.05, 0.08)	0.01 (-0.05, 0.07)	-0.02 (-0.08, 0.05)	<-0.001^ (-0.07, -0.06)	-0.03 (-0.10, 0.03)	0.02 (-0.04, 0.09)
	Multipara	-0.02 (-0.16, 0.12)	-0.11 (-0.31, 0.10)	-0.06 (-0.23, 0.10)	-0.06 (-0.21, 0.09)	-0.03 (-0.19, 0.14)	-0.04 (-0.20, 0.12)
	$P_{interaction}$	0.78	0.26	0.71	0.24	0.82	0.78

^, >-0.01 and <-0.001

HDL, high-density lipoprotein cholesterol; LDL, low-density lipoprotein cholesterol.

Supplementary table 10. Sensitivity analysis of fetal growth at different growth stages and cord blood metabolic factors by excluding women with gestational hypertension

Periods	Growth variable	Regression coefficients (95% confidence intervals)					
		Insulin	Glucose	Total cholesterol	Triglyceride	HDL	LDL
<22 weeks	Weight	0.09 (0.04, 0.14)	0.02 (-0.04, 0.07)	0.02 (-0.03, 0.07)	-0.03(-0.09, 0.02)	0.03 (-0.02, 0.08)	-0.01 (-0.06, 0.04)
	AC	0.10 (0.04, 0.15)	0.02 (-0.03, 0.08)	0.01 (-0.05, 0.06)	-0.04(-0.09, 0.02)	0.03 (-0.02, 0.08)	-0.02 (-0.08, 0.03)
	FL	0.06 (0.01, 0.11)	0.08 (0.03, 0.14)	0.04 (-0.02, 0.09)	-0.02(-0.08, 0.03)	0.09 (0.03, 0.14)	-0.02 (-0.07, 0.03)
22-27 weeks	Weight	0.10 (0.04, 0.15)	0.02 (-0.04, 0.07)	0.02 (-0.03, 0.07)	-0.03(-0.09, 0.02)	0.03 (-0.02, 0.08)	-0.01 (-0.06, 0.05)
	AC	0.11 (0.06, 0.16)	0.03 (-0.03, 0.08)	0.01 (-0.04, 0.06)	-0.04(-0.10, 0.10)	0.03 (-0.02, 0.09)	-0.02 (-0.07, 0.04)
	FL	0.06 (0.01, 0.12)	0.08 (0.03, 0.13)	0.04 (-0.01, 0.09)	-0.02(-0.08, 0.03)	0.09 (0.04, 0.14)	-0.02 (-0.07, 0.04)
28-36 weeks	Weight	0.31 (0.23, 0.39)	0.04 (-0.04, 0.12)	0.05 (-0.03, 0.13)	-0.32(-0.40, -0.24)	0.13 (0.05, 0.21)	0.02 (-0.06, 0.10)
	AC	0.19 (0.13, 0.25)	0.03 (-0.03, 0.09)	0.05 (-0.01, 0.11)	-0.14(-0.20, 0.08)	0.08 (0.03, 0.14)	0.03 (-0.03, 0.08)
	FL	0.04 (-0.01, 0.10)	-0.01 (-0.07, 0.05)	0.05 (-0.01, 0.11)	-0.05(-0.11, 0.01)	0.06 (<-0.001^, 0.11)	-0.05 (-0.01, 0.11)
≥37 weeks	Weight	0.15 (0.10, 0.20)	-0.04 (-0.09, 0.02)	0.01 (-0.04, 0.07)	-0.30 (-0.32, -0.21)	0.04 (-0.01, 0.10)	0.03 (-0.02, 0.09)
	AC	0.06 (<-0.001^, 0.11)	-0.02 (-0.08, 0.04)	-0.01 (-0.07, 0.45)	-0.08(-0.13, -0.02)	0.01 (-0.05, 0.06)	-0.01 (-0.07, 0.04)
	FL	0.02 (-0.04, 0.08)	-0.01 (-0.07, 0.05)	-0.02 (-0.08, 0.04)	<-0.001^ (-0.06, 0.06)	-0.03 (-0.09, 0.03)	0.01 (-0.05, 0.07)

*, >0 and <0.001

^, >-0.01 and <-0.001

HDL, high-density lipoprotein cholesterol; LDL, low-density lipoprotein cholesterol.

AC, abdominal circumference; FL, femur length

Supplementary table 11. Sensitivity analysis of fetal growth at different growth stages and cord blood metabolic factors by excluding preterm infants

Periods	Growth variable	Regression coefficients (95% confidence intervals)					
		Insulin	Glucose	Total cholesterol	Triglyceride	HDL	LDL
<22 weeks	Weight	0.10 (0.05, 0.16)	0.01 (-0.04, 0.06)	0.02 (-0.03, 0.07)	-0.04 (-0.10, 0.01)	0.03 (-0.02, 0.08)	<0.01(-0.05, 0.05)
	AC	0.11 (-0.05, 0.16)	0.02 (-0.04, 0.07)	0.01 (-0.04, 0.06)	-0.05 (-0.10, <0.01)	0.03 (-0.02, 0.09)	-0.02 (-0.07, 0.04)
	FL	0.06 (0.01, 0.11)	0.08 (0.03, 0.14)	0.05 (-0.01, 0.10)	-0.03 (-0.08, 0.02)	0.09 (0.04, 0.14)	-0.01 (-0.06, 0.04)
22-27 weeks	Weight	0.11 (0.06, 0.16)	0.01 (-0.04, 0.06)	0.02 (-0.03, 0.07)	-0.04 (-0.10, 0.01)	0.03 (-0.02, 0.08)	<0.01 (-0.05, 0.05)
	AC	0.12 (0.07, 0.17)	0.02 (-0.03, 0.08)	0.02 (-0.04, 0.07)	-0.05 (-0.11, <0.001^)	0.04 (-0.02, 0.09)	-0.01 (-0.06, 0.04)
	FL	0.06 (0.01, 0.12)	0.08 (0.03, 0.14)	0.05 (<0.01*, 0.10)	-0.02 (-0.08, 0.03)	0.10 (0.04, 0.15)	-0.01 (-0.06, 0.04)
28-36 weeks	Weight	0.31 (0.23, 0.39)	0.04 (-0.05, 0.12)	0.05 (-0.03, 0.13)	-0.33 (-0.40, -0.25)	0.14 (0.06, 0.22)	0.01 (-0.07, 0.09)
	AC	0.19 (0.14, 0.25)	0.03 (-0.03, 0.09)	0.04 (-0.01, 0.10)	-0.14 (-0.20, -0.08)	0.10 (0.04, 0.15)	0.01 (-0.05, 0.07)
	FL	0.04 (-0.01, 0.10)	-0.01 (-0.07, 0.05)	0.06 (<0.01, 0.11)	-0.05 (-0.10, 0.01)	0.06 (<0.01*, 0.12)	0.04 (-0.02, 0.10)
≥37 weeks	Weight	0.15 (0.10, 0.20)	-0.04 (-0.09, 0.02)	<0.01* (-0.05, 0.05)	-0.26(-0.31, 0.21)	0.03 (-0.03, 0.08)	0.02 (-0.03, 0.08)
	AC	0.06 (0.002, 0.11)	-0.02 (-0.07, 0.04)	-0.02 (-0.07, 0.04)	-0.08 (-0.13, -0.02)	<0.001^ (0.06, 0.05)	-0.01 (-0.07, 0.04)
	FL	0.02 (-0.04, 0.07)	-0.01 (-0.07, 0.05)	-0.03 (-0.09, 0.03)	-0.01 (-0.07, 0.05)	-0.04 (-0.10, 0.02)	0.01 (-0.05, 0.07)

*, >0 and <0.001

^, >-0.01 and <-0.001

HDL, high-density lipoprotein cholesterol; LDL, low-density lipoprotein cholesterol.

AC, abdominal circumference; FL, femur length
